# A molybdenum tris­(di­thiol­ene) complex coordinates to three bound cobalt centers in three different ways

**DOI:** 10.1107/S2056989019010363

**Published:** 2019-07-26

**Authors:** Neilson Nguyen, Alan J. Lough, Ulrich Fekl

**Affiliations:** a Department of Chemical and Physical Sciences, University of Toronto Mississauga, 3359 Mississauga Rd, Mississauga, Ontario, L5L 1C6, Canada; b Department of Chemistry, University of Toronto, 80 St. George St., Toronto, Ontario, M5S 3H6, Canada

**Keywords:** crystal structure, ligand, di­thiol­ene, metalloligand

## Abstract

The synthesis and structural characterization of the mol­ecular compound (μ_3_-benzene-1,2-di­thiol­ato)hexa­carbonyl­bis­(μ_3_-1,1,1,4,4,4-hexafluorobut-2-ene-2,3-dithiolato)tricobaltmolybdenum, [Co_3_Mo(C_4_F_6_S_2_)_2_(C_6_H_4_S_2_)(CO)_6_] or Mo(tfd)_2_(bdt)(Co(CO)_2_)_3_ (tfd is 1,1,1,4,4,4-hexafluorobut-2-ene-2,3-dithiolate and bdt is benzene-1,2-di­thiol­ate), are reported. The structure of the mol­ecule contains the molybdenum tris­(di­thiol­ene) complex Mo(tfd)_2_(bdt) coordinated as a multidentate ligand to three cobalt dicarbonyl units. Each of the three cobalt centers is relatively close to molybdenum, with Co⋯Mo distances of 2.7224 (7), 2.8058 (7), and 2.6320 (6) Å. Additionally, each of the cobalt centers is bound *via* main-group donor atoms, but each one in a different way: the first cobalt atom is coordinated by two sulfur atoms from different di­thiol­enes (bdt and tfd). The second cobalt atom is coordinated by one sulfur from one tfd and two olefinic carbons from another tfd. The third cobalt is coordinated by one sulfur from bdt and two sulfurs from tfd. This is, to the best of our knowledge, the first structurally characterized example of a molybdenum (tris­)di­thiol­ene complex that coordinates to cobalt. The F atoms of two of the –CF_3_ groups were refined as disordered over two sets of sites with ratios of refined occupancies of 0.703 (7):0.297 (7) and 0.72 (2):0.28 (2).

## Abstract 2

A molybdenum tris­(di­thiol­ene) complex acts as a ligand towards three Co(CO)_2_ units.

## Chemical context   

Sulfur removal from crude petroleum is performed on a large industrial scale through a process called hydro­desulfurization. This involves use of hydrogen gas (the sulfur is removed as H_2_S) and addition of a catalyst, typically cobalt-doped MoS_2_ (Hinnemann *et al.*, 2008 ▸). MoS_2_ is not a mol­ecular compound but rather possesses an extended structure, consisting of close-packed sulfur layers between which molybdenum is sandwiched (Dickinson & Pauling, 1923 ▸). The coordination geometry around molybdenum is trigonal prismatic. Several attempts to model MoS_2_ using mol­ecular compounds have been made, often using di­thiol­ene (S_2_C_2_ *R*_2_) ligands, where some examples also contain the hydro­desulfurization-relevant addition of cobalt. Complexes containing molybdenum, cobalt, and one or two (but not three) di­thiol­enes are known. A molybdenum bis­(di­thiol­ene) that coordinates to two cobalt centers has been characterized crystallographically (Nihei *et al.*, 1999 ▸; Murata *et al.*, 2006 ▸). The study reports [Mo(bdt)_2_(CO)_2_(CpCo)_2_], where bdt = *o*-C_6_H_4_S_2_ and Cp = *cyclo*-C_5_H_5_. The methyl­ated analog [Mo(bdt)_2_(CO)_2_(Cp*Co)_2_], where Cp* = *cyclo*-C_5_Me_5_ was also structurally characterized (Muratsugu *et al.*, 2011 ▸). An analogous [Mo(ddds)_2_(CO)_2_(CpCo)_2_] was reported by a different group, where ddds is the unusual di­thiol­ene 1,2-dicarba-*closo*-dodeca­borane-1,2-di­sulfide (Chen *et al.*, 2007 ▸). The above contribution also reported a molybdenum mono(di­thiol­ene) complex coordinated to a cobalt fragment, namely [Mo(ddds)(CO)_2_(py)_2_(Cp*Co)], where py = pyridine. Coordinating to cobalt a molybdenum **tris**(di­thioene), that is a compound where three di­thiol­enes are bound to molybdenum, would be inter­esting, because molybdenum tris­(di­thiol­ene)s mimic MoS_2_ particularly well. Similar to MoS_2_, they contain molybdenum coordinated to six sulfur atoms, and, also, depending on the oxidation state of the compound, the environment of molybdenum can sometimes be trigonal prismatic (Beswick *et al.*, 2004 ▸). However, we could not find any structurally characterized example for how a molybdenum tris­(di­thiol­ene) complex can act as ligand for cobalt. Such an example is provided here. In 2007, the mixed di­thiol­ene complex Mo(tfd)_2_(bdt) [tfd = S_2_C_2_(CF_3_)_2_], an unsymmetrical tris­(di­thiol­ene), was reported for the first time (Harrison *et al.*, 2007 ▸). Later, this complex, which contains two different di­thiol­enes, was used to create structural models for the active sites of MoS_2_ hydro­desulfurization catalysts, albeit cobalt-free ones (Nguyen *et al.*, 2010 ▸). In this current work, we have successfully linked three cobalt centers to one Mo(tfd)_2_(bdt) mol­ecule. Surprisingly, each of the three cobalt-containing units [each one is a Co(CO)_2_ fragment] is bound to the molybdenum tris­(di­thiol­ene) center in a different way.
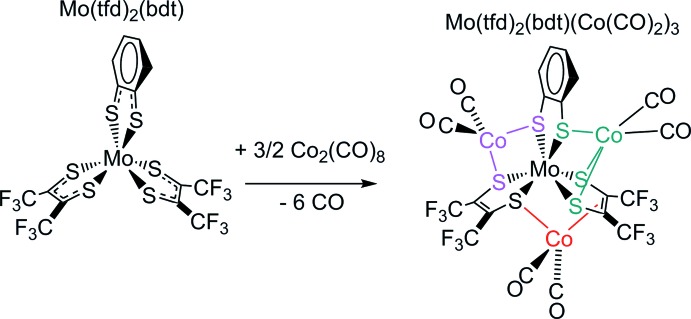


## Structural commentary   

**Figure 1:**
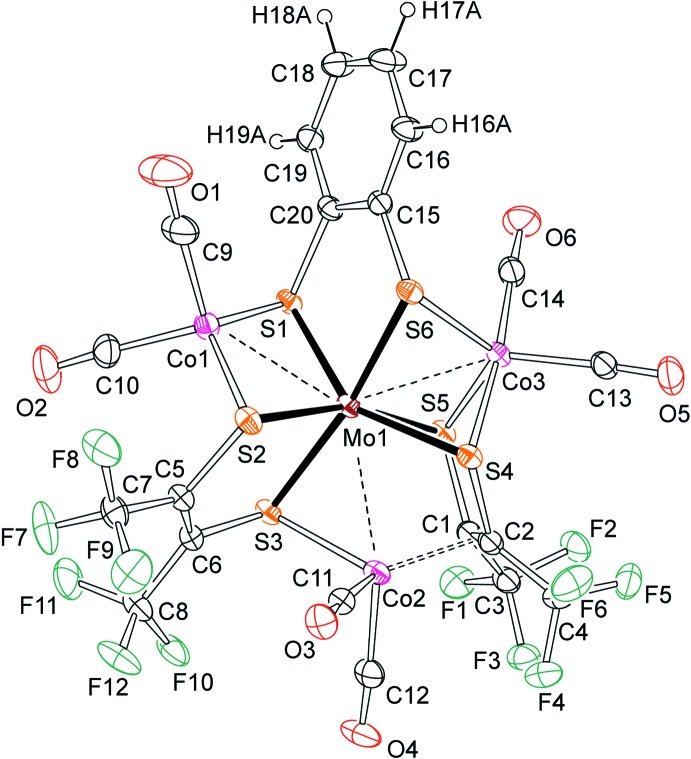
A view of the mol­ecular structure of Mo(tfd)_2_(bdt)(Co(CO)_2_)_3_. Anisotropic displacement ellipsoids in this plot, generated with *ORTEP-3* (Farrugia, 2012 ▸), are shown at the 30% level. Hydrogen atoms are shown as spheres of arbitrary radius. For the disordered fluorines on C7 and C8 only one orientation (major component) is shown.

An anisotropic displacement plot showing the structure of the Mo(tfd)_2_(bdt)(Co(CO)_2_)_3_ mol­ecule is shown in Fig. 1 ▸. A good starting point for the structural description is considering the core Mo(tfd)_2_(bdt) substructure first. Molybdenum is coordinated by six sulfur atoms, four from the two tfd ligands, two from the one bdt ligand. The Mo—S distances are fairly normal, ranging from 2.413 (1) Å (Mo1—S5) to 2.457 (1) Å (Mo1—S4). The appearance of the structure is ‘approximately octa­hedral’. A more qu­anti­tative measure is obtained using the *X*—*M*—*X_trans_* criterion (Beswick *et al.*, 2004 ▸; Nguyen *et al.*, 2010 ▸), which indicates that the geometry around molybdenum is 71% octa­hedral (29% trigonal prismatic). The intra-ring C—C distance in the tfd ligand that is not π-coordinated to cobalt (C5—C6) is 1.335 (6) Å, indicating that description as an ene-di­thiol­ate is appropriate (Hosking *et al.*, 2009 ▸). The intra-ring C-C distance in the tfd ligand that is π-coordinated to cobalt (C1—C2) is much longer, at 1.439 (6) Å, but this elongation is expected as an effect of π-coordination to cobalt (Co2). At this point it makes sense to discuss the way in which the Mo(tfd)_2_(bdt) substructure coordinates to the three cobalt dicarbonyl fragments. Co1 is coordinated by two sulfurs from different di­thiol­enes (bdt and tfd) at bond lengths of 2.225 (1) Å (Co1—S1) and 2.241 (1) Å (Co1—S2), respect­ively. The C_2_S_2_ environment of Co1 is nearly tetra­hedral, with very slight distortions. The S1—Co1—S2 angle is slightly wide, at 115.44 (5)°, the C9—Co1—C10 angle is slightly narrow, at 98.4 (3)°. Co2, in contrast, is coordinated by one sulfur from one tfd and two olefinic carbons from another tfd, where bond lengths are 2.256 (1) Å (Co2—S3), 2.010 (4) Å (Co2—C1), and 1.970 (4) (Co2—C2). The coordination geometry of Co2 (not including Mo1 here) is, again, approximately tetra­hedral, where the largest deviation from tetra­hedral geometry are C11—Co2—C12, at 96.3 (2)° and the comparably wide ‘bite’ of the chelating substructure, with *Ct*1—Co2—S3 measuring 119.7°, where *Ct*1 is the mid-point between C11 and C12. Another sulfur atom, S4, is relatively close to Co2, but the inter­atomic distance, at 2.767 (1) Å, is considerably longer than the Co2—S3 bond, such that S4 is almost certainly not bonded. Finally, Co3 is coordinated by one sulfur from bdt and two sulfurs from tfd, at distances of 2.234 (1) Å (Co3—S4), 2.239 (1) Å (Co3—S5), and 2.275 (1) Å (Co3—S6). Co3 is surrounded by these three sulfurs and two carbons in an approximately trigonal–bipyramidal fashion. C14 and S4 occupy axial positions, with the C14-Co3-S4 angle being 174.3 (2)°. The three angles in the trigonal plane are 115.38 (4)° (S5—Co3—S6), 118.6 (2)° (S5—Co3—C13), and 124.0 (2)° (S6—Co3—C13). While there is no doubt that the three cobalt atoms are bound by the heteroatoms (sulfur, carbon) of the Mo(tfd)_2_(bdt) structure, each of the three cobalt atoms is also close to the central molybdenum, and these metal–metal contacts could possibly be bonding as well. The relevant distances are 2.7224 (7) Å (Co1—Mo1), 2.8058 (7) Å (Co2—Mo1), and 2.6320 (6) Å (Co3—Mo1). Such Mo—Co distances are typically considered of a range compatible with Mo—Co bonds (Chen *et al.*, 2007 ▸; Curtis *et al.*, 1997 ▸; Murata *et al.*, 2006 ▸; Muratsugu *et al.*, 2011 ▸).

## Supra­molecular features   

**Figure 2:**
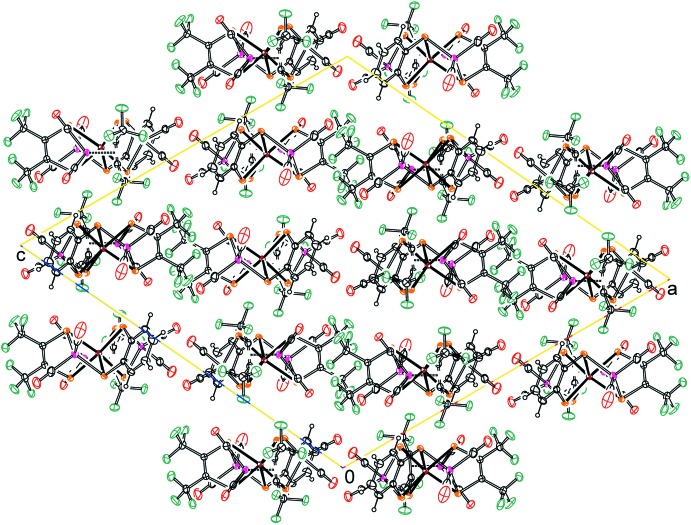
Packing of mol­ecules of Mo(tfd)_2_(bdt)(Co(CO)_2_)_3_, viewed along the *b* axis.

Mol­ecules of Mo(tfd)_2_(bdt)(Co(CO)_2_)_3_ pack, without any solvent in the crystal, *via* contacting van der Waals surfaces. The packing pattern is shown in Fig. 2 ▸. Hydrogen atom H17*A* forms close inter­molecular contacts to an oxygen atom from a neighboring carbonyl and to a fluorine atom of the major disorder component (F11), as well as to a fluorine atom of the minor disorder component (F10*A*). Details can be found in Table 1 ▸.

## Database survey   

The Cambridge Crystallographic Database (version 5.40, including updates up to May 2019; Groom *et al.*, 2016 ▸) was searched. The search was performed as a substructure search containing the most general di­thiol­ene S–C–C–S substructure (with any kind of bond allowed in the chain), plus a molybdenum and a cobalt atom. Since no specific requirement was imposed with regard to whether or in which way cobalt or molybdenum are bonded to the S–C–C–S structure, hits that do not contain a molybdenum di­thiol­ene complex coordinated to cobalt where manually removed as follows. Seven hits were retrieved: EYUHIQ, JOQWIV, JOQWIV01, SEVMIQ, SEVMOW, TASDAT, OQAMEZ. Out of these, TASDAT and OQAMEZ are not relevant here, since they do not contain a molybdenum di­thiol­ene unit that is directly bonded to cobalt. They contain, respectively, a cobalt-based counter-cation for an anionic molybdenum complex and a nickel bis­(di­thiol­ene) anion as a counter-anion for a molybdenum/cobalt sulfido cluster. The structures EYUHIQ, JOQWIV, JOQWIV01, SEVMIQ and SEVMOW are relevant, since they contain at least one molybdenum di­thiol­ene unit that is directly bonded to cobalt. These structures are all discussed above in the *Chemical context* (Nihei *et al.*, 1999 ▸; Murata *et al.*, 2006 ▸; Muratsugu *et al.*, 2011 ▸; Chen *et al.*, 2007 ▸).

## Synthesis and crystallization   

**Figure 3:**
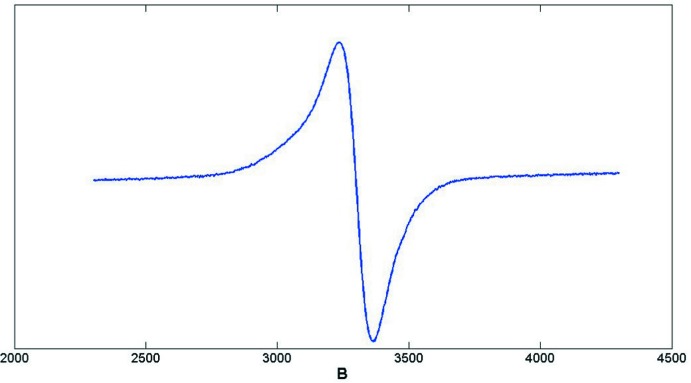
X-band EPR spectrum of Mo(tfd)_2_(bdt)(Co(CO)_2_)_3_ in hexane at 298 K; *g* = 2.010.

Mo(tfd)_2_(bdt)(Co(CO)_2_)_3_ was prepared from Mo(tfd)_2_(bdt) (Harrison *et al.*, 2007 ▸) and dicobaltocta­carbonyl (obtained from Sigma-Aldrich) as summarized in the Scheme, using air-free conditions and rigorously dried solvents. 48 mg of Mo(tfd)_2_(bdt) (0.0697 mmol) were dissolved in 18 mL of hexane (dried over Na/benzo­phenone). 60 mg (0.175 mmol) of (Co)_2_(CO)_8_ dissolved in 2 mL of hexane were added and the mixture was shaken. The mixture was stored overnight at 243 K in the freezer of a nitro­gen-filled glovebox. The supernatant was deca­nted off, and the black crystals of Mo(tfd)_2_(bdt)(Co(CO)_2_)_3_ were washed twice with 5 mL of cold hexane. Total yield 27 mg (0.026 mmol, 37%). Analysis calculated for Mo_1_S_6_C_20_H_4_F_12_Co_3_O_6_: C, 23.25; H, 0.39; O, 9.29; S, 18.62. Found: C, 23.70; H, 0.44; O, 9.70; S, 18.80. ^1^H NMR (400 MHz, C_6_D_6_): δ 6.38 (*m*), 7.02 (*m*). The compound is paramagnetic. An estimate of the magnetic moment in solution (Evans method) yielded *ca* 0.9 BM, consistent with one unpaired electron. An EPR spectrum was also obtained, shown in Fig. 3 ▸.

## Refinement   

Crystal data, data collection and structure refinement details are summarized in Table 2 ▸. H atoms were placed in calculated positions and included in a riding-motion approximation with *U*_iso_(H) = 1.2*U*_eq_(C). The F atoms of the –CF_3_ groups containing C7 and C8 were refined as disordered over two sets of sites with ratios of refined occupancies of 0.703 (7):0.297 (7) and 0.72 (2):0.28 (2), respectively.

## Supplementary Material

Crystal structure: contains datablock(s) I. DOI: 10.1107/S2056989019010363/zl2758sup1.cif

Structure factors: contains datablock(s) I. DOI: 10.1107/S2056989019010363/zl2758Isup3.hkl

CCDC reference: 1941888

Additional supporting information:  crystallographic information; 3D view; checkCIF report

## Figures and Tables

**Table 1:** Hydrogen-bond geometry (Å, °)

*D*—H⋯*A*	*D*—H	H⋯*A*	*D*⋯*A*	*D*—H⋯*A*
C17—H17*A*⋯O6^i^	0.95	2.57	3.353 (7)	140
C17—H17*A*⋯F11^ii^	0.95	2.62	3.461 (9)	148
C17—H17*A*⋯F10*A* ^ii^	0.95	2.55	3.29 (2)	135

Symmetry codes: (i) 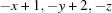; (ii) 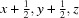.

**Table 2:** Experimental details

Crystal data	
Chemical formula	[Co_3_Mo(C_4_F_6_S_2_)_2_(C_6_H_4_S_2_)(CO)_6_]
*M*_r_	1033.32
Crystal system, space group	Monoclinic, *C*2/*c*
Temperature (K)	150
*a*, *b*, *c* (Å)	22.7465 (5), 12.8779 (5), 23.6033 (6)
β (°)	115.3840 (16)
*V* (Å^3^)	6246.5 (3)
*Z*	8
Radiation type	Mo *K*α
μ (mm^−1^)	2.47
Crystal size (mm)	0.32 × 0.12 × 0.10

Data collection	
Diffractometer	Nonius KappaCCD
Absorption correction	Multi-scan (*SORTAV*; Blessing, 1995 ▸)
*T*_min_, *T*_max_	0.686, 0.798
No. of measured, independent and observed [*I* > 2σ(*I*)] reflections	21250, 7102, 5019
*R*_int_	0.046
(sin θ/λ)_max_ (Å^−1^)	0.650

Refinement	
*R*[*F* ^2^ > 2σ(*F* ^2^)], *wR*(*F* ^2^), *S*	0.045, 0.112, 1.07
No. of reflections	7102
No. of parameters	489
No. of restraints	210
H-atom treatment	H-atom parameters constrained
Δρ_max_, Δρ_min_ (e Å^−3^)	1.09, −0.74

Computer programs: *COLLECT* (Nonius, 2002 ▸), *DENZO-SMN* (Otwinowski & Minor, 1997 ▸), *SIR92* (Altomare *et al.*, 1994 ▸), *SHELXL2018* (Sheldrick, 2015 ▸), *PLATON* (Spek, 2009 ▸) and *SHELXTL* (Sheldrick, 2008 ▸).

## supplementary crystallographic information

### Crystal data

**Table:**

[Co_3_Mo(C_4_F_6_S_2_)_2_(C_6_H_4_S_2_)(CO)_6_]	*F*(000) = 3992
*M**_r_* = 1033.32	*D*_x_ = 2.198 Mg m^−^^3^
Monoclinic, *C*2/*c*	Mo *K*α radiation, λ = 0.71073 Å
*a* = 22.7465 (5) Å	Cell parameters from 21250 reflections
*b* = 12.8779 (5) Å	θ = 3.0–27.5°
*c* = 23.6033 (6) Å	µ = 2.47 mm^−^^1^
β = 115.3840 (16)°	*T* = 150 K
*V* = 6246.5 (3) Å^3^	Needle, dark red
*Z* = 8	0.32 × 0.12 × 0.10 mm

### Data collection

**Table:**

Nonius KappaCCD diffractometer	7102 independent reflections
Radiation source: fine-focus sealed tube	5019 reflections with *I* > 2σ(*I*)
Detector resolution: 9 pixels mm^-1^	*R*_int_ = 0.046
φ scans and ω scans with κ offsets	θ_max_ = 27.5°, θ_min_ = 3.0°
Absorption correction: multi-scan (SORTAV; Blessing, 1995)	*h* = −29→29
*T*_min_ = 0.686, *T*_max_ = 0.798	*k* = −16→16
21250 measured reflections	*l* = −30→30

### Refinement

**Table:**

Refinement on *F*^2^	Primary atom site location: structure-invariant direct methods
Least-squares matrix: full	Secondary atom site location: difference Fourier map
*R*[*F*^2^ > 2σ(*F*^2^)] = 0.045	Hydrogen site location: inferred from neighbouring sites
*wR*(*F*^2^) = 0.112	H-atom parameters constrained
*S* = 1.07	*w* = 1/[σ^2^(*F*_o_^2^) + (0.054*P*)^2^ + 5.1797*P*] where *P* = (*F*_o_^2^ + 2*F*_c_^2^)/3
7102 reflections	(Δ/σ)_max_ = 0.001
489 parameters	Δρ_max_ = 1.09 e Å^−^^3^
210 restraints	Δρ_min_ = −0.74 e Å^−^^3^

### Special details

**Table:**

Geometry. All esds (except the esd in the dihedral angle between two l.s. planes) are estimated using the full covariance matrix. The cell esds are taken into account individually in the estimation of esds in distances, angles and torsion angles; correlations between esds in cell parameters are only used when they are defined by crystal symmetry. An approximate (isotropic) treatment of cell esds is used for estimating esds involving l.s. planes.

### Fractional atomic coordinates and isotropic or equivalent isotropic displacement parameters (Å^2^)

**Table:**

	*x*	*y*	*z*	*U*_iso_*/*U*_eq_	Occ. (<1)
Mo1	0.37011 (2)	0.67610 (3)	0.12477 (2)	0.02586 (11)	
Co1	0.32882 (3)	0.85549 (5)	0.15702 (3)	0.03548 (16)	
Co2	0.33267 (3)	0.47117 (5)	0.13309 (3)	0.03083 (16)	
Co3	0.45749 (3)	0.63936 (5)	0.08184 (3)	0.02962 (15)	
S1	0.32447 (5)	0.82941 (9)	0.06212 (5)	0.0297 (2)	
S2	0.36985 (5)	0.72385 (9)	0.22479 (5)	0.0331 (3)	
S3	0.26312 (5)	0.60667 (9)	0.10435 (5)	0.0296 (2)	
S4	0.45637 (5)	0.54398 (9)	0.16042 (5)	0.0301 (2)	
S5	0.35447 (5)	0.58534 (9)	0.02990 (5)	0.0295 (2)	
S6	0.47364 (5)	0.76648 (9)	0.15395 (5)	0.0307 (3)	
F1	0.26591 (12)	0.4072 (2)	−0.02994 (12)	0.0493 (7)	
F2	0.35969 (14)	0.3736 (2)	−0.02658 (12)	0.0484 (7)	
F3	0.32346 (13)	0.2830 (2)	0.02808 (12)	0.0456 (7)	
F4	0.41197 (15)	0.2522 (2)	0.14259 (14)	0.0581 (8)	
F5	0.46739 (15)	0.3033 (2)	0.09356 (15)	0.0588 (8)	
F6	0.49947 (14)	0.3377 (2)	0.19160 (14)	0.0635 (9)	
F7	0.2282 (2)	0.7313 (7)	0.2705 (2)	0.074 (2)	0.703 (7)
F8	0.3246 (3)	0.7860 (5)	0.3117 (3)	0.0698 (18)	0.703 (7)
F9	0.3064 (4)	0.6287 (4)	0.3225 (2)	0.0766 (19)	0.703 (7)
F7A	0.2573 (8)	0.7953 (11)	0.2754 (6)	0.072 (4)	0.297 (7)
F8A	0.3435 (5)	0.7165 (15)	0.3305 (4)	0.063 (3)	0.297 (7)
F9A	0.2537 (7)	0.6372 (10)	0.2967 (5)	0.063 (3)	0.297 (7)
F10	0.1632 (4)	0.5120 (8)	0.1246 (4)	0.055 (2)	0.72 (2)
F11	0.1431 (3)	0.6580 (6)	0.1565 (6)	0.066 (2)	0.72 (2)
F12	0.1944 (4)	0.5381 (9)	0.2222 (3)	0.068 (2)	0.72 (2)
F10A	0.1475 (9)	0.558 (2)	0.1103 (7)	0.057 (4)	0.28 (2)
F11A	0.1543 (10)	0.6471 (14)	0.1877 (12)	0.056 (4)	0.28 (2)
F12A	0.1972 (10)	0.4990 (14)	0.2037 (10)	0.057 (4)	0.28 (2)
O1	0.3951 (3)	1.0559 (4)	0.2075 (3)	0.112 (2)	
O2	0.19536 (19)	0.9067 (4)	0.1404 (2)	0.0771 (13)	
O3	0.37570 (16)	0.4528 (3)	0.26994 (14)	0.0486 (9)	
O4	0.23791 (19)	0.2999 (3)	0.09880 (16)	0.0587 (11)	
O5	0.56008 (17)	0.5074 (3)	0.07720 (17)	0.0586 (10)	
O6	0.4473 (2)	0.7725 (3)	−0.02293 (17)	0.0610 (10)	
C1	0.35680 (19)	0.4568 (3)	0.06092 (19)	0.0304 (10)	
C2	0.41017 (19)	0.4338 (4)	0.1204 (2)	0.0323 (10)	
C3	0.3269 (2)	0.3798 (4)	0.0090 (2)	0.0372 (11)	
C4	0.4467 (2)	0.3306 (4)	0.1367 (2)	0.0423 (12)	
C5	0.29505 (19)	0.6793 (4)	0.22259 (18)	0.0309 (10)	
C6	0.25188 (19)	0.6280 (3)	0.17312 (19)	0.0304 (10)	
C7	0.2880 (2)	0.7059 (4)	0.2817 (2)	0.0437 (12)	
C8	0.1880 (2)	0.5842 (4)	0.1692 (2)	0.0435 (12)	
C9	0.3691 (3)	0.9805 (5)	0.1880 (3)	0.0646 (17)	
C10	0.2465 (3)	0.8898 (4)	0.1474 (2)	0.0495 (13)	
C11	0.3593 (2)	0.4622 (4)	0.2178 (2)	0.0354 (10)	
C12	0.2757 (2)	0.3625 (4)	0.1124 (2)	0.0409 (11)	
C13	0.5214 (2)	0.5607 (4)	0.0791 (2)	0.0370 (11)	
C14	0.4513 (2)	0.7229 (4)	0.0182 (2)	0.0410 (11)	
C15	0.45531 (19)	0.8839 (3)	0.11004 (19)	0.0296 (9)	
C16	0.5057 (2)	0.9503 (4)	0.1170 (2)	0.0398 (11)	
H16A	0.549037	0.934569	0.146045	0.048*	
C17	0.4928 (2)	1.0394 (4)	0.0814 (2)	0.0512 (14)	
H17A	0.527525	1.083894	0.085070	0.061*	
C18	0.4294 (2)	1.0649 (4)	0.0401 (2)	0.0442 (12)	
H18A	0.420821	1.127055	0.016168	0.053*	
C19	0.3788 (2)	0.9995 (4)	0.0339 (2)	0.0347 (10)	
H19A	0.335466	1.016576	0.005600	0.042*	
C20	0.39134 (19)	0.9089 (3)	0.06908 (19)	0.0291 (9)	

### Atomic displacement parameters (Å^2^)

**Table:**

	*U*^11^	*U*^22^	*U*^33^	*U*^12^	*U*^13^	*U*^23^
Mo1	0.02129 (18)	0.0321 (2)	0.02433 (19)	−0.00421 (15)	0.00990 (15)	−0.00087 (15)
Co1	0.0354 (3)	0.0368 (4)	0.0387 (3)	−0.0013 (3)	0.0202 (3)	−0.0033 (3)
Co2	0.0325 (3)	0.0345 (4)	0.0287 (3)	−0.0072 (3)	0.0162 (3)	−0.0013 (2)
Co3	0.0259 (3)	0.0351 (4)	0.0308 (3)	−0.0016 (3)	0.0149 (2)	0.0027 (3)
S1	0.0216 (5)	0.0358 (6)	0.0311 (5)	−0.0030 (4)	0.0107 (4)	0.0019 (5)
S2	0.0278 (5)	0.0441 (7)	0.0269 (5)	−0.0067 (5)	0.0112 (4)	−0.0048 (5)
S3	0.0242 (5)	0.0394 (7)	0.0261 (5)	−0.0077 (5)	0.0117 (4)	−0.0025 (5)
S4	0.0286 (5)	0.0344 (6)	0.0283 (5)	−0.0017 (5)	0.0130 (4)	0.0029 (5)
S5	0.0275 (5)	0.0357 (6)	0.0269 (5)	−0.0036 (5)	0.0133 (4)	−0.0001 (5)
S6	0.0229 (5)	0.0362 (7)	0.0304 (5)	−0.0045 (4)	0.0088 (4)	0.0008 (5)
F1	0.0445 (15)	0.0509 (18)	0.0423 (15)	−0.0106 (14)	0.0088 (13)	−0.0117 (13)
F2	0.0626 (17)	0.0540 (19)	0.0453 (15)	−0.0151 (15)	0.0391 (14)	−0.0152 (13)
F3	0.0604 (17)	0.0358 (16)	0.0460 (15)	−0.0124 (13)	0.0281 (13)	−0.0079 (12)
F4	0.0690 (19)	0.0357 (17)	0.074 (2)	−0.0050 (15)	0.0343 (17)	0.0094 (15)
F5	0.073 (2)	0.0481 (19)	0.072 (2)	0.0172 (16)	0.0474 (18)	0.0034 (15)
F6	0.0556 (19)	0.0473 (19)	0.0605 (19)	0.0085 (15)	−0.0008 (16)	0.0074 (15)
F7	0.043 (3)	0.130 (6)	0.055 (3)	0.008 (3)	0.027 (2)	−0.032 (4)
F8	0.092 (4)	0.085 (4)	0.054 (3)	−0.038 (4)	0.051 (3)	−0.038 (3)
F9	0.124 (5)	0.081 (4)	0.033 (3)	0.019 (4)	0.041 (3)	0.012 (3)
F7A	0.097 (8)	0.077 (8)	0.052 (6)	0.022 (7)	0.042 (6)	−0.017 (6)
F8A	0.061 (6)	0.106 (9)	0.028 (5)	−0.005 (7)	0.025 (5)	−0.006 (6)
F9A	0.083 (8)	0.081 (7)	0.038 (6)	−0.025 (7)	0.038 (5)	−0.011 (5)
F10	0.045 (4)	0.069 (5)	0.063 (4)	−0.029 (3)	0.035 (3)	−0.024 (3)
F11	0.030 (3)	0.084 (4)	0.084 (5)	0.006 (2)	0.024 (3)	−0.006 (4)
F12	0.062 (3)	0.099 (5)	0.053 (3)	−0.032 (4)	0.035 (3)	0.005 (4)
F10A	0.031 (6)	0.087 (10)	0.052 (7)	−0.023 (7)	0.017 (5)	−0.012 (7)
F11A	0.044 (7)	0.067 (7)	0.072 (9)	−0.008 (6)	0.039 (7)	−0.017 (7)
F12A	0.055 (6)	0.065 (8)	0.063 (8)	−0.014 (7)	0.036 (6)	0.005 (7)
O1	0.183 (5)	0.076 (4)	0.116 (4)	−0.071 (4)	0.102 (4)	−0.047 (3)
O2	0.058 (2)	0.107 (4)	0.082 (3)	0.038 (3)	0.045 (2)	0.027 (3)
O3	0.053 (2)	0.063 (3)	0.0311 (18)	0.0045 (18)	0.0197 (16)	0.0060 (16)
O4	0.078 (3)	0.063 (3)	0.051 (2)	−0.042 (2)	0.043 (2)	−0.0207 (19)
O5	0.052 (2)	0.068 (3)	0.069 (2)	0.020 (2)	0.0381 (19)	0.012 (2)
O6	0.091 (3)	0.053 (2)	0.054 (2)	−0.002 (2)	0.045 (2)	0.0113 (19)
C1	0.031 (2)	0.030 (2)	0.034 (2)	−0.0034 (19)	0.0177 (19)	−0.0025 (19)
C2	0.033 (2)	0.034 (3)	0.035 (2)	−0.0041 (19)	0.0195 (19)	−0.0018 (19)
C3	0.039 (3)	0.041 (3)	0.037 (2)	−0.007 (2)	0.022 (2)	−0.003 (2)
C4	0.048 (3)	0.032 (3)	0.047 (3)	−0.007 (2)	0.021 (2)	0.000 (2)
C5	0.027 (2)	0.039 (3)	0.028 (2)	−0.0028 (19)	0.0126 (18)	0.0002 (19)
C6	0.027 (2)	0.036 (3)	0.031 (2)	0.0008 (19)	0.0146 (19)	0.0010 (19)
C7	0.044 (3)	0.061 (3)	0.032 (2)	−0.001 (3)	0.022 (2)	−0.006 (2)
C8	0.036 (2)	0.059 (3)	0.044 (3)	−0.010 (2)	0.024 (2)	−0.002 (2)
C9	0.098 (5)	0.056 (4)	0.065 (4)	−0.029 (4)	0.059 (4)	−0.022 (3)
C10	0.058 (3)	0.049 (3)	0.052 (3)	0.010 (3)	0.034 (3)	0.003 (3)
C11	0.036 (2)	0.034 (3)	0.039 (3)	−0.001 (2)	0.019 (2)	0.000 (2)
C12	0.053 (3)	0.046 (3)	0.032 (2)	−0.009 (3)	0.026 (2)	−0.008 (2)
C13	0.034 (2)	0.042 (3)	0.040 (3)	0.003 (2)	0.020 (2)	0.009 (2)
C14	0.039 (3)	0.045 (3)	0.045 (3)	−0.001 (2)	0.024 (2)	−0.005 (2)
C15	0.029 (2)	0.032 (3)	0.030 (2)	−0.0031 (18)	0.0144 (19)	−0.0032 (18)
C16	0.026 (2)	0.040 (3)	0.048 (3)	−0.008 (2)	0.011 (2)	0.003 (2)
C17	0.045 (3)	0.042 (3)	0.064 (3)	−0.017 (2)	0.021 (3)	0.005 (3)
C18	0.043 (3)	0.035 (3)	0.050 (3)	−0.004 (2)	0.016 (2)	0.009 (2)
C19	0.029 (2)	0.036 (3)	0.037 (2)	−0.002 (2)	0.012 (2)	−0.003 (2)
C20	0.026 (2)	0.034 (3)	0.029 (2)	−0.0074 (18)	0.0129 (18)	−0.0057 (19)

### Geometric parameters (Å, º)

**Table:**

Mo1—S5	2.4134 (11)	F6—C4	1.339 (5)
Mo1—S1	2.4179 (11)	F7—C7	1.312 (6)
Mo1—S3	2.4391 (10)	F8—C7	1.324 (6)
Mo1—S2	2.4420 (11)	F9—C7	1.321 (6)
Mo1—S6	2.4461 (11)	F7A—C7	1.322 (10)
Mo1—S4	2.4571 (11)	F8A—C7	1.300 (10)
Mo1—Co3	2.6320 (6)	F9A—C7	1.324 (9)
Mo1—Co1	2.7224 (7)	F10—C8	1.333 (7)
Mo1—Co2	2.8058 (7)	F11—C8	1.332 (7)
Co1—C10	1.840 (5)	F12—C8	1.336 (7)
Co1—C9	1.842 (6)	F10A—C8	1.341 (12)
Co1—S1	2.2247 (12)	F11A—C8	1.312 (12)
Co1—S2	2.2414 (13)	F12A—C8	1.329 (12)
Co2—C12	1.825 (5)	O1—C9	1.127 (7)
Co2—C11	1.827 (5)	O2—C10	1.125 (6)
Co2—C2	1.970 (4)	O3—C11	1.130 (5)
Co2—C1	2.010 (4)	O4—C12	1.122 (5)
Co2—S3	2.2556 (13)	O5—C13	1.131 (5)
Co2—S4	2.7671 (11)	O6—C14	1.133 (6)
Co3—C13	1.796 (5)	C1—C2	1.439 (6)
Co3—C14	1.803 (5)	C1—C3	1.494 (6)
Co3—S4	2.2338 (12)	C2—C4	1.528 (7)
Co3—S5	2.2395 (11)	C5—C6	1.335 (6)
Co3—S6	2.2749 (13)	C5—C7	1.509 (6)
S1—C20	1.782 (4)	C6—C8	1.524 (6)
S2—C5	1.775 (4)	C15—C16	1.383 (6)
S3—C6	1.770 (4)	C15—C20	1.396 (6)
S4—C2	1.777 (4)	C16—C17	1.377 (7)
S5—C1	1.801 (4)	C16—H16A	0.9500
S6—C15	1.779 (4)	C17—C18	1.390 (7)
F1—C3	1.341 (5)	C17—H17A	0.9500
F2—C3	1.344 (5)	C18—C19	1.381 (6)
F3—C3	1.339 (5)	C18—H18A	0.9500
F4—C4	1.324 (5)	C19—C20	1.389 (6)
F5—C4	1.340 (6)	C19—H19A	0.9500
			
S5—Mo1—S1	88.52 (4)	C2—S4—Co3	102.26 (15)
S5—Mo1—S3	84.34 (4)	C2—S4—Mo1	99.62 (14)
S1—Mo1—S3	92.75 (4)	Co3—S4—Mo1	68.07 (3)
S5—Mo1—S2	163.43 (4)	C2—S4—Co2	45.16 (13)
S1—Mo1—S2	101.96 (4)	Co3—S4—Co2	111.42 (4)
S3—Mo1—S2	82.39 (4)	Mo1—S4—Co2	64.65 (3)
S5—Mo1—S6	103.47 (4)	C1—S5—Co3	102.78 (13)
S1—Mo1—S6	83.85 (4)	C1—S5—Mo1	95.79 (14)
S3—Mo1—S6	171.36 (4)	Co3—S5—Mo1	68.78 (3)
S2—Mo1—S6	90.52 (4)	C15—S6—Co3	104.69 (14)
S5—Mo1—S4	76.17 (4)	C15—S6—Mo1	106.48 (13)
S1—Mo1—S4	147.71 (4)	Co3—S6—Mo1	67.65 (3)
S3—Mo1—S4	113.36 (4)	C2—C1—C3	124.1 (4)
S2—Mo1—S4	100.13 (4)	C2—C1—S5	116.8 (3)
S6—Mo1—S4	72.66 (4)	C3—C1—S5	110.6 (3)
S5—Mo1—Co3	52.48 (3)	C2—C1—Co2	67.3 (2)
S1—Mo1—Co3	96.23 (3)	C3—C1—Co2	124.2 (3)
S3—Mo1—Co3	135.41 (3)	S5—C1—Co2	106.8 (2)
S2—Mo1—Co3	137.11 (3)	C1—C2—C4	124.4 (4)
S6—Mo1—Co3	53.08 (3)	C1—C2—S4	114.4 (3)
S4—Mo1—Co3	51.93 (3)	C4—C2—S4	115.3 (3)
S5—Mo1—Co1	137.65 (3)	C1—C2—Co2	70.3 (2)
S1—Mo1—Co1	50.87 (3)	C4—C2—Co2	126.8 (3)
S3—Mo1—Co1	86.08 (3)	S4—C2—Co2	95.1 (2)
S2—Mo1—Co1	51.09 (3)	F3—C3—F1	106.5 (3)
S6—Mo1—Co1	85.61 (3)	F3—C3—F2	106.4 (4)
S4—Mo1—Co1	144.34 (3)	F1—C3—F2	106.0 (3)
Co3—Mo1—Co1	131.93 (2)	F3—C3—C1	114.4 (4)
S5—Mo1—Co2	71.40 (3)	F1—C3—C1	111.2 (4)
S1—Mo1—Co2	138.35 (3)	F2—C3—C1	111.9 (4)
S3—Mo1—Co2	50.35 (3)	F4—C4—F6	106.2 (4)
S2—Mo1—Co2	92.44 (3)	F4—C4—F5	107.4 (4)
S6—Mo1—Co2	135.42 (3)	F6—C4—F5	107.0 (4)
S4—Mo1—Co2	63.03 (3)	F4—C4—C2	113.9 (4)
Co3—Mo1—Co2	99.20 (2)	F6—C4—C2	110.4 (4)
Co1—Mo1—Co2	128.84 (2)	F5—C4—C2	111.5 (4)
C10—Co1—C9	98.4 (3)	C6—C5—C7	126.2 (4)
C10—Co1—S1	107.95 (16)	C6—C5—S2	121.5 (3)
C9—Co1—S1	109.32 (18)	C7—C5—S2	112.3 (3)
C10—Co1—S2	111.11 (17)	C5—C6—C8	124.2 (4)
C9—Co1—S2	113.2 (2)	C5—C6—S3	122.6 (3)
S1—Co1—S2	115.44 (5)	C8—C6—S3	113.3 (3)
C10—Co1—Mo1	128.61 (18)	F7—C7—F9	108.1 (5)
C9—Co1—Mo1	132.8 (2)	F8A—C7—F7A	105.8 (10)
S1—Co1—Mo1	57.46 (3)	F7—C7—F8	105.8 (5)
S2—Co1—Mo1	57.97 (3)	F9—C7—F8	105.4 (5)
C12—Co2—C11	96.3 (2)	F8A—C7—F9A	107.0 (9)
C12—Co2—C2	110.5 (2)	F7A—C7—F9A	105.6 (10)
C11—Co2—C2	104.60 (18)	F8A—C7—C5	113.3 (6)
C12—Co2—C1	97.04 (18)	F7—C7—C5	112.5 (4)
C11—Co2—C1	146.99 (18)	F9—C7—C5	112.2 (4)
C2—Co2—C1	42.40 (16)	F7A—C7—C5	110.7 (6)
C12—Co2—S3	100.76 (17)	F8—C7—C5	112.3 (4)
C11—Co2—S3	103.81 (15)	F9A—C7—C5	113.8 (6)
C2—Co2—S3	134.51 (14)	F11A—C8—F12A	105.5 (11)
C1—Co2—S3	103.14 (13)	F11—C8—F10	107.2 (5)
C12—Co2—S4	148.89 (16)	F11—C8—F12	107.0 (6)
C11—Co2—S4	86.51 (14)	F10—C8—F12	105.6 (6)
C2—Co2—S4	39.76 (13)	F11A—C8—F10A	105.2 (11)
C1—Co2—S4	66.97 (12)	F12A—C8—F10A	106.5 (11)
S3—Co2—S4	108.66 (4)	F11A—C8—C6	115.3 (9)
C12—Co2—Mo1	154.70 (16)	F12A—C8—C6	111.9 (9)
C11—Co2—Mo1	99.65 (15)	F11—C8—C6	111.6 (5)
C2—Co2—Mo1	84.29 (13)	F10—C8—C6	112.1 (5)
C1—Co2—Mo1	79.96 (12)	F12—C8—C6	112.9 (5)
S3—Co2—Mo1	56.37 (3)	F10A—C8—C6	111.8 (9)
S4—Co2—Mo1	52.32 (3)	O1—C9—Co1	178.5 (8)
C13—Co3—C14	94.7 (2)	O2—C10—Co1	177.0 (6)
C13—Co3—S4	91.03 (14)	O3—C11—Co2	177.4 (4)
C14—Co3—S4	174.25 (15)	O4—C12—Co2	176.0 (5)
C13—Co3—S5	118.60 (16)	O5—C13—Co3	176.9 (4)
C14—Co3—S5	92.28 (15)	O6—C14—Co3	177.6 (5)
S4—Co3—S5	84.38 (4)	C16—C15—C20	120.3 (4)
C13—Co3—S6	124.00 (15)	C16—C15—S6	118.8 (3)
C14—Co3—S6	97.07 (16)	C20—C15—S6	120.9 (3)
S4—Co3—S6	80.22 (4)	C17—C16—C15	119.5 (4)
S5—Co3—S6	115.38 (4)	C17—C16—H16A	120.2
C13—Co3—Mo1	150.72 (14)	C15—C16—H16A	120.2
C14—Co3—Mo1	114.26 (15)	C16—C17—C18	120.8 (4)
S4—Co3—Mo1	60.00 (3)	C16—C17—H17A	119.6
S5—Co3—Mo1	58.74 (3)	C18—C17—H17A	119.6
S6—Co3—Mo1	59.27 (3)	C19—C18—C17	119.7 (4)
C20—S1—Co1	98.78 (14)	C19—C18—H18A	120.1
C20—S1—Mo1	106.69 (15)	C17—C18—H18A	120.1
Co1—S1—Mo1	71.67 (4)	C18—C19—C20	120.1 (4)
C5—S2—Co1	97.05 (15)	C18—C19—H19A	120.0
C5—S2—Mo1	106.85 (14)	C20—C19—H19A	120.0
Co1—S2—Mo1	70.94 (3)	C19—C20—C15	119.6 (4)
C6—S3—Co2	101.70 (15)	C19—C20—S1	118.7 (3)
C6—S3—Mo1	106.61 (14)	C15—C20—S1	121.8 (3)
Co2—S3—Mo1	73.29 (3)		
			
Co3—S5—C1—C2	20.2 (3)	Co2—S3—C6—C5	79.7 (4)
Mo1—S5—C1—C2	−49.3 (3)	Mo1—S3—C6—C5	3.9 (4)
Co3—S5—C1—C3	−129.4 (3)	Co2—S3—C6—C8	−101.5 (3)
Mo1—S5—C1—C3	161.1 (3)	Mo1—S3—C6—C8	−177.3 (3)
Co3—S5—C1—Co2	92.78 (16)	C6—C5—C7—F8A	153.6 (10)
Mo1—S5—C1—Co2	23.27 (17)	S2—C5—C7—F8A	−26.0 (11)
C3—C1—C2—C4	4.9 (7)	C6—C5—C7—F7	−37.8 (8)
S5—C1—C2—C4	−140.2 (4)	S2—C5—C7—F7	142.6 (5)
Co2—C1—C2—C4	121.8 (4)	C6—C5—C7—F9	84.4 (7)
C3—C1—C2—S4	156.5 (3)	S2—C5—C7—F9	−95.2 (5)
S5—C1—C2—S4	11.4 (4)	C6—C5—C7—F7A	−87.7 (11)
Co2—C1—C2—S4	−86.6 (3)	S2—C5—C7—F7A	92.7 (10)
C3—C1—C2—Co2	−116.9 (4)	C6—C5—C7—F8	−157.0 (6)
S5—C1—C2—Co2	98.0 (3)	S2—C5—C7—F8	23.4 (6)
Co3—S4—C2—C1	−37.5 (3)	C6—C5—C7—F9A	31.1 (11)
Mo1—S4—C2—C1	32.0 (3)	S2—C5—C7—F9A	−148.5 (9)
Co2—S4—C2—C1	70.6 (3)	C5—C6—C8—F11A	45.7 (14)
Co3—S4—C2—C4	116.8 (3)	S3—C6—C8—F11A	−133.1 (14)
Mo1—S4—C2—C4	−173.7 (3)	C5—C6—C8—F12A	−74.8 (13)
Co2—S4—C2—C4	−135.1 (4)	S3—C6—C8—F12A	106.4 (13)
Co3—S4—C2—Co2	−108.11 (14)	C5—C6—C8—F11	78.1 (8)
Mo1—S4—C2—Co2	−38.65 (16)	S3—C6—C8—F11	−100.7 (7)
C2—C1—C3—F3	36.6 (6)	C5—C6—C8—F10	−161.6 (7)
S5—C1—C3—F3	−176.6 (3)	S3—C6—C8—F10	19.6 (8)
Co2—C1—C3—F3	−47.6 (5)	C5—C6—C8—F12	−42.4 (9)
C2—C1—C3—F1	157.2 (4)	S3—C6—C8—F12	138.8 (7)
S5—C1—C3—F1	−55.9 (4)	C5—C6—C8—F10A	165.8 (14)
Co2—C1—C3—F1	73.1 (5)	S3—C6—C8—F10A	−13.0 (14)
C2—C1—C3—F2	−84.5 (5)	Co3—S6—C15—C16	−108.6 (3)
S5—C1—C3—F2	62.4 (4)	Mo1—S6—C15—C16	−179.2 (3)
Co2—C1—C3—F2	−168.6 (3)	Co3—S6—C15—C20	71.1 (4)
C1—C2—C4—F4	−69.7 (6)	Mo1—S6—C15—C20	0.5 (4)
S4—C2—C4—F4	138.9 (3)	C20—C15—C16—C17	−2.5 (7)
Co2—C2—C4—F4	20.4 (6)	S6—C15—C16—C17	177.3 (4)
C1—C2—C4—F6	170.9 (4)	C15—C16—C17—C18	2.0 (8)
S4—C2—C4—F6	19.5 (5)	C16—C17—C18—C19	−0.9 (8)
Co2—C2—C4—F6	−99.0 (4)	C17—C18—C19—C20	0.3 (7)
C1—C2—C4—F5	52.1 (6)	C18—C19—C20—C15	−0.8 (7)
S4—C2—C4—F5	−99.3 (4)	C18—C19—C20—S1	177.5 (4)
Co2—C2—C4—F5	142.3 (3)	C16—C15—C20—C19	1.9 (6)
Co1—S2—C5—C6	73.6 (4)	S6—C15—C20—C19	−177.9 (3)
Mo1—S2—C5—C6	1.4 (4)	C16—C15—C20—S1	−176.3 (3)
Co1—S2—C5—C7	−106.8 (3)	S6—C15—C20—S1	3.9 (5)
Mo1—S2—C5—C7	−178.9 (3)	Co1—S1—C20—C19	−111.0 (3)
C7—C5—C6—C8	−2.0 (7)	Mo1—S1—C20—C19	175.6 (3)
S2—C5—C6—C8	177.6 (3)	Co1—S1—C20—C15	67.2 (4)
C7—C5—C6—S3	176.7 (4)	Mo1—S1—C20—C15	−6.2 (4)
S2—C5—C6—S3	−3.7 (6)		

### Hydrogen-bond geometry (Å, º)

**Table:**

*D*—H···*A*	*D*—H	H···*A*	*D*···*A*	*D*—H···*A*
C17—H17*A*···O6^i^	0.95	2.57	3.353 (7)	140
C17—H17*A*···F11^ii^	0.95	2.62	3.461 (9)	148
C17—H17*A*···F10*A*^ii^	0.95	2.55	3.29 (2)	135

Symmetry codes: (i) −*x*+1, −*y*+2, −*z*; (ii) *x*+1/2, *y*+1/2, *z*.
